# Multi-protein spatial signatures in ductal carcinoma in situ (DCIS) of breast

**DOI:** 10.1038/s41416-020-01216-6

**Published:** 2021-01-07

**Authors:** Sunil S. Badve, Sanghee Cho, Yesim Gökmen-Polar, Yunxia Sui, Chrystal Chadwick, Elizabeth McDonough, Anup Sood, Marian Taylor, Maria Zavodszky, Puay Hoon Tan, Michael Gerdes, Adrian L. Harris, Fiona Ginty

**Affiliations:** 1 Department of Pathology and Laboratory Medicine, Indiana University School of Medicine, Indianapolis, IN 46202 USA; 2 GE Research, Niskayuna, NY 12309 USA; 3 Department of Oncology, Cancer and Haematology Centre, Oxford University, Oxford, OX37LJ UK; 4 Department of Pathology, Singapore General Hospital, Singapore, Singapore

**Keywords:** Breast cancer, Prognostic markers

## Abstract

**Background:**

There is limited knowledge about DCIS cellular composition and relationship with breast cancer events (BCE).

**Methods:**

Immunofluorescence multiplexing (MxIF) was used to image and quantify 32 cellular biomarkers in FFPE DCIS tissue microarrays. Over 75,000 DCIS cells from 51 patients (median 9 years follow-up for non-BCE cases) were analysed for profiles predictive of BCE. K-means clustering was used to evaluate cellular co-expression of epithelial markers with ER and HER2.

**Results:**

Only ER, PR and HER2 significantly correlated with BCE. Cluster analysis identified 6 distinct cell groups with different levels of ER, Her2, cMET and SLC7A5. Clusters 1 and 3 were not significant. Clusters 2 and 4 (high ER/low HER2 and SLC7A5/mixed cMET) significantly correlated with low BCE risk (*P* = 0.001 and *P* = 0.034), while cluster 6 (high HER2/low ER, cMET and SLC7A5) correlated with increased risk (*P* = 0.018). Cluster 5 (similar to cluster 6, except high SLC7A5) trended towards significance (*P* = 0.072). A continuous expression score (Escore) based on these 4 clusters predicted likelihood of BCE (AUC = 0.79, log-rank test *P* = 5E–05; LOOCV AUC = 0.74, log-rank test *P* = 0.006).

**Conclusion:**

Multiplexed spatial analysis of limited tissue is a novel method for biomarker analysis and predicting BCEs. Further validation of Escore is needed in a larger cohort.

## Background

The successful implementation of the breast screening program in developed countries has resulted in the identification of a large number of putative precursor lesions of invasive carcinoma. Ductal carcinoma in situ (DCIS) is a non-obligate precursor lesion that is managed aggressively. Most patients get treated with surgery followed by post-operative radiation therapy. These therapies have been documented to decrease the incidence of recurrence and development of invasive cancer. In addition, the UK/ANZ DCIS trial and the NSABP B-24 clinical trials further demonstrated a significant reduction in frequency of DCIS recurrence by the addition of endocrine therapy with resultant recurrence rates below 10%.^1–3^

DCIS, if left untreated, will progress to invasive carcinoma in only around 20–50% of patients.^4–6^ This has led to significant concerns regarding overtreatment of patients. Currently, there are many trials that are enrolling patients with DCIS for non-surgical management based on histological features of DCIS. Low risk DCIS cases are being enrolled in the LORIS trial in the United Kingdom, LORD trial in Europe and LARRIKIN trial in Australia for non-surgical management by active surveillance.^7–9^ This is similar to the COMET (comparing operative to monitoring and endocrine therapy for low risk DCIS) trial in the USA.^10^ Inclusion criteria of the COMET Trial include women 40 years or older with screen-detected calcification associated with histologically confirmed low-grade (LG) or intermediate-grade (IG) DCIS. The LORIS Trial and LORD Trial also study screen-detected calcification associated with LG or IG DCIS. However, there are some differences in age of eligibility, whether patients with bilateral or multicentric disease, prior breast disease or mantle radiation can be enrolled. The presence of comedo-necrosis is an important exclusion criterion in some of these trials. These histological features are subjective and there is poor inter-observer agreement due to intratumoural heterogeneity.^11–13^ A recent survey of more than 30 international recognised breast pathologists documented marked variability in definition of comedo-necrosis.^14^ This subjectivity will significantly impact patient enrolment and final study results. There is a clear need for better understanding the biology of DCIS and the pathways leading to (or associated with) progression.

A number of tools have been used for the prognostication of DCIS. These include histological features, single marker, as well as multi-marker panels for immunohistochemistry assays, in addition to multiplex RT-qPCR for mRNAs. Analysis of the 12-year follow-up data of the Eastern Cooperative Oncology Group (ECOG) E 5194 trial has confirmed the role of histological features (high grade) in predicting likelihood of recurrence.^15^ This was also confirmed in analysis of 57,222 DCIS cases from the SEER database by Sagara et al.^16^ The expression of oestrogen and progesterone receptors (ER and PR) is also associated with decreased risk of recurrence. In contrast, proliferation markers such as Ki67 are associated with a higher risk (reviewed in Groen et al.^9^). The Tlsty group has analysed the expression of p16, COX2 and Ki67 to identify an IHC based predictor for the likelihood of recurrence.^17^ In a multivariable model, DCIS lesions that were p16^+^/COX2^+^/Ki67^+^ or those detected by palpation were statistically significantly associated with subsequent invasive cancer.^17^ Based on these initial analyses, they have identified a panel of IHC biomarkers (PR, HER2, Ki67, COX2, p16/INK4A, FOXA1 and SIAH2), which is commercially available through Prelude’s CLIA-approved lab as DCIS on RT™ (Decision score (DS)). In multivariable analysis, DS, but not nuclear grade, correlated with the benefit of radiotherapy in the SweDCIS cohort.^18^ In collaboration with Genomic Health Inc, we have described a 12-gene signature for DCIS.^19^ This signature is based on mRNA levels of progesterone (*PgR*), Glutathione S-Transferase Mu 1 (*GSTM1*) and 5 proliferation genes (*MKI67, STK15, BIRC5, CCNB1*, and *MYBL2*). This has been further validated in independent studies, including the Toronto cohort.^20^ We have also recently used multiplexed immunofluorescence imaging (MxIF) to study the degree of protein heterogeneity in DCIS.^13^ This study documented marked heterogeneity at a single cell level in DCIS. One of the limitations of the study was the lack of clinical follow-up data, which limited understanding the impact of the heterogeneity in DCIS.

The current exploratory study was designed to further understand the impact of a large number of biomarkers previously reported to be associated with second breast cancer event (BCE) of DCIS using a cohort of well annotated DCIS cases with follow-up data. These included ER, PR, HER2, Ki67, p53, COX2, and CD10 which have been well described in DCIS. We further investigated the HER pathway by analysing EGFR, HER4 and PTEN. We also investigated the role of cancer stem cells (ALDH1 and CD44v6),^21^ and proteins implicated in progression (p21, VEGFR2, cMET, CDCP1, HTF9C/TRMT2A, and CEACAM5)^22–26^ and resistance to therapy (ABCB1, ABCG2, MRP4, MRP5, SLC7A5).^27–30^ DAPI was used to identify the nuclei and together with NaKATPase, pan-cytokeratin and S6 used for epithelial cell segmentation. Multiplexed immunofluorescence and single cell analysis^13,31^ was used to analyse the (co-) expression of these markers in a single FFPE TMA sections.

## Methods

### Clinical Cohort

IRB permissions were obtained from Oxford University (for the entire study) and waiver of IRB from Indiana University. De-identified DCIS cases were selected from the archives at Oxford University/Radcliffe General Hospital. The selection criteria were as follows: (1) they were excision specimens; (2) patients did not have invasive (or microinvasive) cancer; (3) patients did not have any prior therapy; (4) patients did not have prior history of breast cancer. The patients were diagnosed between 1986 and 2004 and selected on the basis of a long follow-up period, with a median of 8 years (range 1–17 years). This was to allow evaluation of the long natural history of DCIS biology. An initial histopathological review was performed to confirm the diagnosis of DCIS, which was confirmed independently by two pathologists.

Of the 135 patients, 62 were excluded due to insufficient clinical information or poor data quality, leaving 73 patients with follow-up data. As will be described in the ‘Results’ section, a further 17 patients were not included in the analysis due to insufficient or no DCIS in the sectioned tissue. The final number of patients was 51 (13 BCEs and 38 non-BCE). Demographic and treatment data are summarised for all patients (*N* = 135) and the final group used for outcome analysis (*N* = 51) in Supplementary Table 1.

Breast cancer screening was introduced into the UK in 1988, for women aged between 50 and 70 years, so many of the younger patients in this study presented symptomatically. Patients were subsequently treated off-protocol with lumpectomy (80%) or mastectomy (20%), adjuvant hormone therapy (56%), and adjuvant radiotherapy (74%) and 16% had both radiotherapy and endocrine therapy (Tamoxifen). None of the patients received aromatase inhibitors.

After the review of H&Es, tissue microarrays were constructed from 135 patients, 2 mm dimension, with 40 cores per slide and 8 clinical slides total including 1 replicate, plus one control array with a mix of breast cancer and DCIS cores. Patients were primarily Caucasian, with age range of 34–75 (median age 55 years). The primary endpoint was any breast cancer event including ipsilateral or contralateral breast events and in rare cases metastases.

### Multiplexed immunofluorescence imaging of DCIS TMAs

Multiplexed immunofluorescence iterative staining of the DCIS TMAs was performed as previously described^13,31^ using the Cell DIVE™ technology (Cytiva. Issaquah, WA). Briefly, slides were rehydrated, underwent a two-step antigen retrieval and were stained using a Leica Bond autostainer. After de-paraffinisation, and antigen retrieval,^31^ the slides were incubated with antibodies at manufacturer recommended concentrations for 1 h at room temperature. All antibodies were validated per the protocol described previously.^13,31^ Where possible, antibodies used in a clinical IHC lab were included in the screenings. After selection, each antibody was conjugated with either Cy3, Cy5 or Cy7 bis-NHS-ester dyes using standard protocols as previously described.^31^ Supplementary Table 2 shows the antibodies, clones and conjugates used in the study.

All the sections underwent multiplexed staining for 32 markers (staining for one marker (S6) was repeated due to poor staining in the second round) using antibodies at concentrations as listed in Supplementary Table 3. Briefly the 32 markers and staining rounds were as follows: Round 1: CK5/6, Her4; Round 2: ABCG2, PTEN, S6; Round 3: CD20, S6 (repeated), CKAE1; Round 4: PR, ER, NaKATPAse; Round 5: CK19, ALDH1, PCK26; Round 6: CD4, cMET; Round 7: CD44v6, HER2; Round 8: CDCP1, p53; Round 9: CK15, Cox2; Round 10: VEGFR2, ABCB1; Round 11: HTF9c, CD10; Round 12: MRP4, SLC7A5; Round 13: EGFR, p21; Round 14: MRP5, CEACAM5; Round 15 Ki67 (note that in total, 6 background imaging rounds were also included). Example stains and FOV for all markers are shown in Supplementary Fig. 1. All biomarkers stained as expected and were included in univariate analysis, however, staining for EGFR was very weak in some cases and was excluded from later analyses.

### Image processing, single cell segmentation

**Fig. 1:**
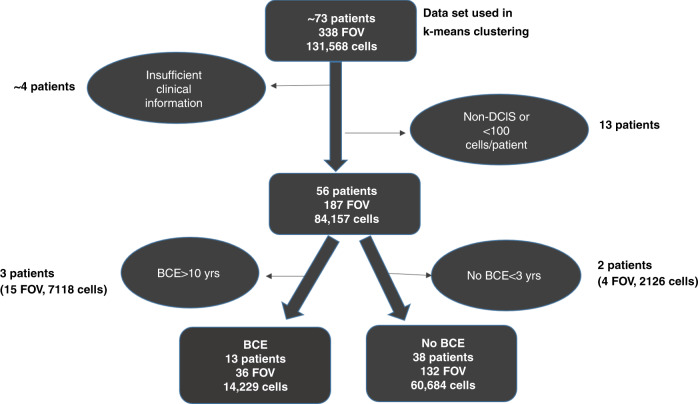
A flow chart representing the total number of cells and fields of view (FOV) that were analysed and the filtering process that was used for breast cancer events (BCE) analysis. The end result of this process led to the analysis of 51 patients (13 patients with BCEs and 38 without BCEs).

Cells in the epithelial and stromal compartments were then segmented using DAPI, pan-cytokeratin, S6, and NaKATPase, as previously described.^13,31^ Several quality control steps were then conducted, including visual review and manual scoring of tissue quality and segmentation for every image. Images with poor quality staining or too few cells or lacking DCIS histology were excluded from data analysis. Also, additional cell filtering was conducted on the segmented images using the following criteria: (1) epithelial cells were required to have 1–2 number of nuclei; (2) each sub-cellular compartment (nucleus, membrane, cytoplasm) area had to have >10 pixels and <1500 pixels; and (3) cells in each round of staining had to have excellent alignment with the first round of staining (QC score = 1). For this, an automated QC score was generated for every cell in each imaging round by correlating baseline DAPI images with all corresponding DAPI images from other multiplexing rounds. A perfect score of 1 indicated perfect registration, no cell loss and no cell movement. A score of 0 indicated complete loss of that cell after baseline imaging. Supplementary Table 4 provides details of all the QC steps and the final number of cells that underwent statistical analysis. After the QC steps, the data underwent exposure time correction, log2 transformation to handle the skewness of the marker intensities, and normalisation to remove any slide batch effects. Additional patient level filtering was also applied as shown in Fig. 1 to ensure a final dataset with complete clinical data. Median nuclear intensity was used for ER, PR, p21, Ki67 and median intensity of the whole cell for the remaining markers.

### Statistical analysis

Correlation analysis was conducted for all biomarkers to determine co-expression patterns. For univariate and multivariate outcome analysis, the primary clinical endpoint was development of a BCE. Benjamini–Hochberg procedure with a false discovery rate 0.2 was applied to determine statistical significance of the association with BCE. For outcome analysis, images with insufficient DCIS content (%DCIS/(%DCIS + %Normal) <0.5), and patients with less than 100 cells were excluded. Also, patients who were reported as having non-BCE, but had a follow-up time less than 3 years were excluded. Similarly, those who were reported as having BCE, but ≥10 years were also excluded to remove the possibility of a second primary.

In the first step of univariate analysis, violin plots of every biomarker distribution in the BCE and non-BCE groups were generated. Mean of the median cell intensities per marker were used for patient level aggregation and *t*-test was used to evaluate the mean differences between groups.

Unsupervised k-means clustering was applied with number of group k = 2, …, 15 for all markers and subsets of markers. Extreme values (1% on both tails) were capped and standardised with zero mean and single standard deviation to remove unit effect of each marker. Consensus clustering^32^ was used to determine the best number of distinct clusters. Consensus clustering involves repeating k-means clustering in a subset of the data and measuring how consistently the data separates into groups. PAC (proportion of ambiguously clustered)^33^ and a visual check of the heatmap were used to evaluate the cluster separation. Following cluster analysis, the proportion of cells in each cluster, was determined for each patient (patient cluster profile). The patient cluster profile was evaluated in multivariate logistic regression models to determine probability of BCE for a given patient cluster profile. Leave-one-out cross validation was also performed to confirm the results. Log-rank test and Kaplan–Meier plot were generated to evaluate the effectiveness of the model.

## Results

### Generation of a multi-step analyses workflow for the development of a DCIS breast cancer event score using multiplexed cell analysis data

As described in the ‘Methods’ section, the study cohort consisted of 13 patients who had a breast cancer event (BCE) within 10 years (median 2.5 years), and 38 patients who did not develop BCE within 3–10 years (median 9 years) (Supplementary Table 1). Survival analysis of the entire cohort compared to the study cohort showed no difference in outcome (Supplementary Fig. 2).

To develop a risk score for DCIS cases from multiplexed tissue, we developed a comprehensive multi-step data analyses and cell filtering workflow, as shown in Supplementary Table 3. Briefly, of the ~2 million cell-like objects (which included non-cell objects), which were segmented using the previously described membrane, cytoplasm and nuclear markers, 40% were classified as potential epithelial cells based on their location within cytokeratin positive tissue regions. After applying the filtering criteria described in the method section, 131,568 nucleated epithelial cells were used for clustering analysis. Of those, 74,913 cells with matching clinical data were used for outcome analysis.

**Fig. 2:**
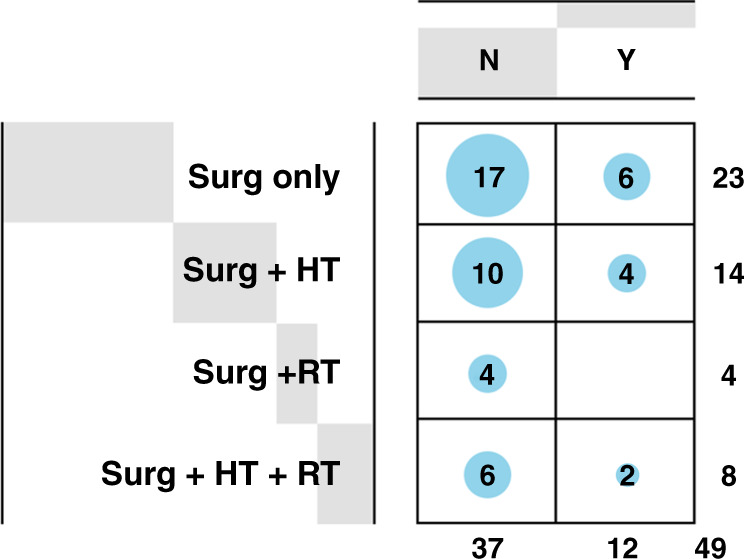
Characteristics of the clinical cohort. Incidence of breast cancer events (BCEs) in patients with hormone therapy (HT), radiotherapy (RT), or surgery (Surg) alone or in combination. N-No BCE, Y-Yes for BCE.

Clinical features such as patient age and menopause status and histological features such as grade and comedonecrosis were not associated with breast cancer events (BCE). Further, no difference in treatment regimens were found between the non-BCE and BCE groups (Fig. 2) and thus treatment was not included in the final model.

Example images for all markers are shown in Supplementary Fig. 1. Univariate correlation analysis plots between all markers, irrespective of group, in Supplementary Fig. 3. To initially analyse the association between marker expression and BCE, violin plots were generated for each of the markers, shown in Supplementary Fig. 4. In univariate analysis, higher HER2 and lower ER and PR expression were associated with BCE (based on Benjamini–Hochberg procedure with a false discovery rate 0.2). EGFR was associated with BCE but was excluded from the analysis due to overall poor and inconsistent staining quality. The remainder of the markers that were not associated with BCE were as follows: ABCB1, ABCG2, ALDH1, CDCP1, CD10, CD44v6, CEACAM5, CK-15, CK-19, CK-56, CK-AE1, CK-PCK26, cMET, HER4, HTF9C, MRP4, PTEN, MRP5, NaKATPase, p53, p21, S6, SLC7A5 and VEGFR2. Notably, Ki67 and COX2 were not associated with BCEs (*P* = 0.561 and *P* = 0.851, respectively).

### K-Means clustering of cellular protein expression

Based on the initial univariate results, we conducted cell cluster analysis with subsets of all epithelial biomarkers along with HER2 and ER to explore which biomarkers were co-expressed with this clinical phenotype and significantly correlated with outcome (PR was excluded as it was highly correlated with ER expression). Although neither SLC7A5 nor cMET were independently correlated with BCE in univariate analysis, when clustered along with ER and HER2 positive cells they were consistently aligned. Using consensus clustering of ER, HER2, SLC7A5 and cMET, 6 clusters with varying levels of each marker were found to provide the best separation.

**Fig. 3:**
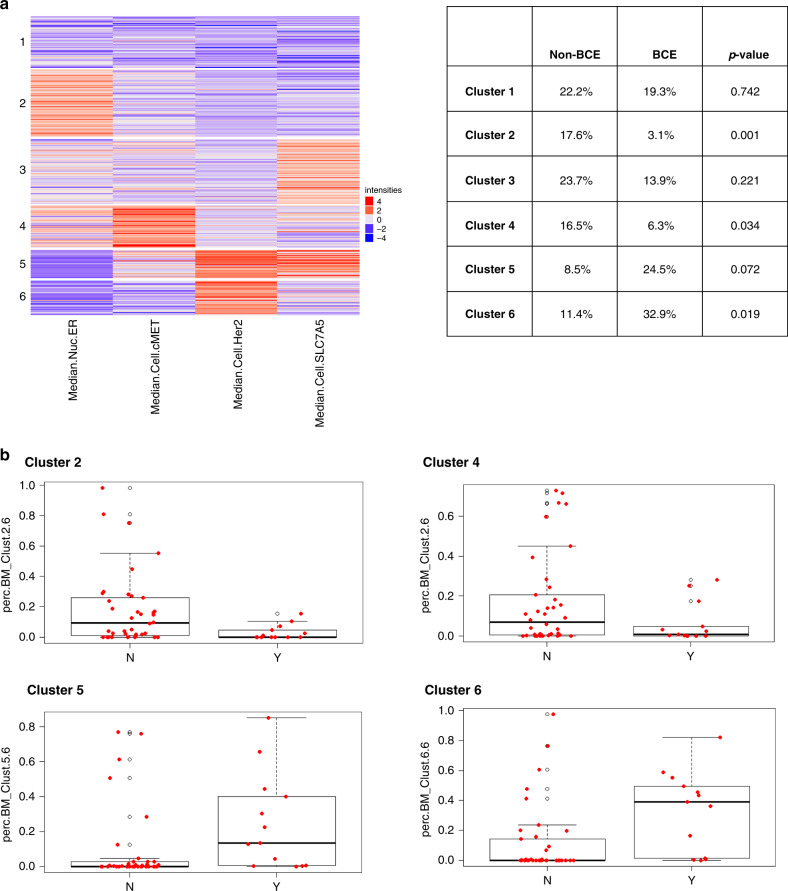
Contribution of cell clusters to BCE and non-BCE. **a** Heatmap depicting the results of the unsupervised k-means clustering. The use of four markers (HER2, SLC7A5, ER, and cMET6) resulted in the identification of six clusters (1–6). Cluster 2 cells were enriched for ER positivity, and cluster 4 shows ER and cMET positivity. HER2 expression was observed in both clusters 5 and 6. The likelihood of breast cancer events (BCE) associated with each of the clusters is also shown. **b** Box plots showing the association of the clusters with likelihood of BCE. BCE patients tended to have very little cluster 2 and 4 type cells and more of  cluster 5 and 6 type cells. After k-means clustering is performed, the proportion of each cell type (clusters) in each patient is calculated. Comparison of cluster profiles by BCE showed that cluster 2 and 4 cells appeared more in non-BCE patients while cluster 5 and 6 appeared more in BCE patients.

**Fig. 4:**
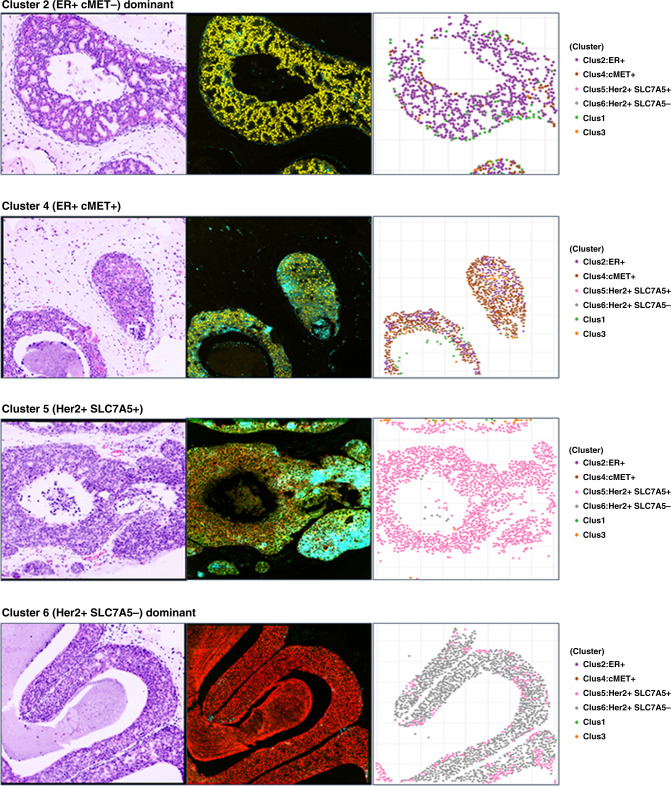

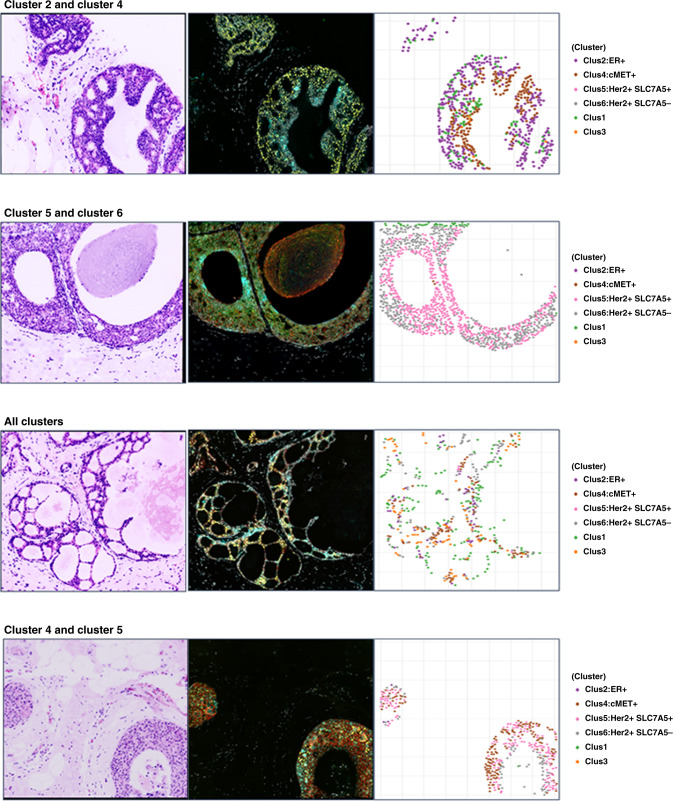
Virtual H&E images for each example cluster, biomaker stains and cluster plot of the cells expressing those markers at different levels of intensity. Ducts can either show dominant expression of a single cluster or mixed expression pattern of clusters.

Figure 3a shows the cluster distribution heatmap for all 6 clusters and a breakdown of cluster profiles for the BCE and non-BCE groups is shown in the adjacent table. As shown in Fig. 3b, cluster 2, characterised by higher expression of ER and lower HER2, SLC7A5 and cMET, accounted for 17.6% of cells in the non-BCE group and was strongly associated with lack of BCE (*P* = 0.001), compared to 3% of cluster 2 cells in the BCE group. Similarly, cluster 4 (higher expression of ER and cMET, and lower HER2 and SLC7A5) accounted for 16.5% of cells in the non-BCE group and was also associated with lack of BCE (*P* = 0.034), vs 6.3% in the BCE group. Cluster 6 with higher expression of HER2, lower SLC7A5, ER and cMET, accounted for 33% of cells in the BCE group and was associated with high risk of BCE (*P* = 0.018), vs 11.4% of cells in the non-BCE group. Cluster 5, with both high HER2 and SLC7A5, low-moderate cMET and low ER accounted for 24.5% of cells in the BCE group (vs. 8.5% in the non-BCE group), associated with high risk of BCE (*P* = 0.072), but suggesting a possible impact of SLC7A5 in determination of BCE. Example images including virtual H&E, biomarker stains and associated cluster plots for each cluster and combinations of clusters are shown in Fig. 4a, b. Cluster 1, which had low expression for all 4 markers or Cluster 3, which had high SLC7A5 and low HER2 were not significantly correlated with the outcome (*P* = 0.742 and 0.221, respectively).

### Development of logistic regression-based algorithm for predicting risk of BCE using cell clusters (Escore)

**Fig. 5:**
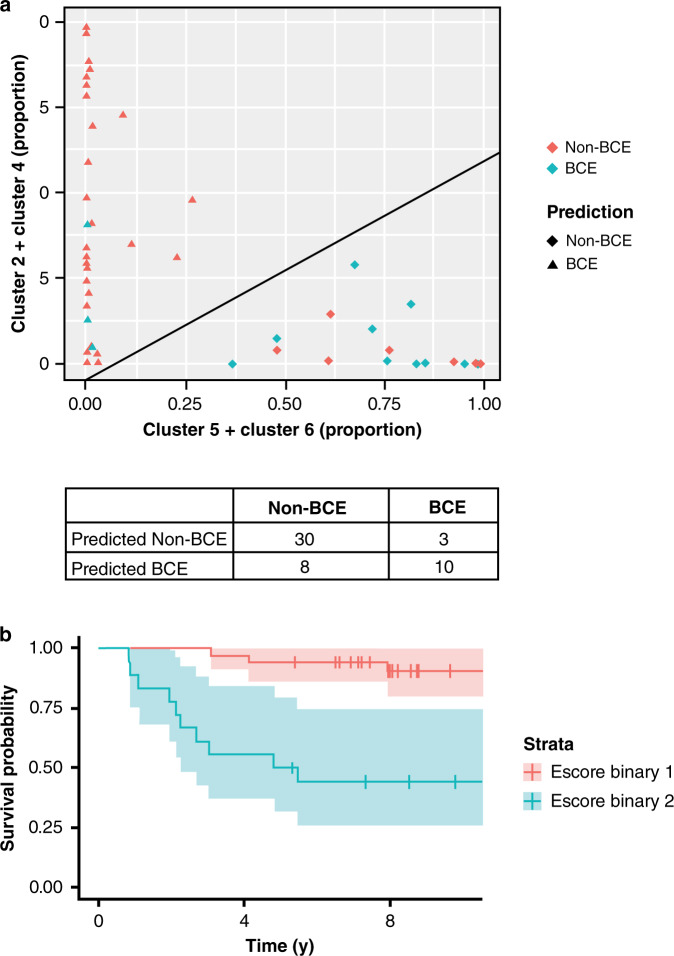
Model performance for prediction of BCE and non-BCE. **a** Classification model was developed using logistic regression to predict BCE and non-BCE using two input variables, one comprising of cluster 2 and 4, and the other comprising of cluster 5 and 6. The model had a sensitivity of 77%, specificity of 79% with an error rate of 21.6% and AUC of 0.785. The AUC in the leave one out cross-validation was 0.739. **b** Escore was developed from the classification model and binary Escore was used for log-rank analysis and data represented using Kaplan–Meier plots.

In order to further assess the clinical utility of the cell clusters, logistic regression analysis was performed using combinations of clusters that were correlated with BCE (clusters 2 + 4 and clusters 5 + 6). The model gave an AUC of 0.79 (0.74 with leave-one-out cross validation). This analysis was further converted into an expression score of the likelihood of BCE, ‘Escore’, based on the predicted probability of BCE from the logistic regression model (Fig. 5a). Escore: 1.77*(%Clus5&6) − 2.78*(%Clus2&4) >13 was the criteria for the high-risk BCE with sensitivity (TPR) of 77% and a specificity (TNR) of 79%. The threshold was based on the predicted probability of BCE being higher than the group BCE rate of 0.255 (13 divided by 51). Figure 5b shows the disease-free-interval analysis using Kaplan–Meier plots for the two groups. As shown in these plots, binary categorisation of the Escore results in clear separation of the survival curves (*P* = 5E–05) with low scores being associated with marked decrease in likelihood of BCE. In initial validation using the leave-one-out cross validation, Escore remained significantly associated with BCE (*P* = 0.006).

The cohort had 10 patients for whom data was available on duplicate cores. Analysis of these cores showed consistent results; none of the patients would have been re-classified based on these additional cores. This suggests that even in this small cohort and limited tissue sample availability, Escore was not affected by heterogeneity; however, the sample size is too small for a definitive conclusion. A recent database update resulted in the reclassification of BCE status of 4 patients from non-BCE to BCE. As these BCEs occurred 9.6–16.2 years after initial diagnosis, these might be potentially new diseases. Two of these 4 patients were in the low risk group, as determined by Escore.

## Discussion

Biological heterogeneity in cancer is well recognised,^12^ leading to the understanding that not all cancers need to be treated aggressively. In the case of invasive breast cancer, gene expression assay-based trials such as the MINDACT (microarray in the determination of adjuvant chemotherapy) and TAILORx (trial assigning Individualised options for treatment Rx) have documented that a significant number of women can safely avoid chemotherapy.^34,35^ Both assays were good at identifying classes of patients that benefit from chemotherapy (high clinical and high molecular risk groups) and that can safely avoid chemotherapy (low clinical and low molecular risk groups).

Epidemiological studies have documented that overall survival rates for DCIS are around 95% at 10 years.^36^ Therefore, it is natural to seek to identify categories of patients for whom therapy can be reduced. Additionally, there is great concern about ‘overdiagnosis’ and hence overtreatment screen detected DCIS.^37^ DCIS has been traditionally treated with surgery followed by hormonal therapy and or radiotherapy to the breast to prevent recurrence of DCIS or development of invasive cancer. The current clinical trials (LORIS, LORD and COMET) are enrolling patients on histological features^7,8,10^; this in part due to lack of good molecular markers. One of the major limitations of the immunohistochemical or mRNA panels is the amount of tissue required for analysis. This is particularly true in cases where important management decisions are going to be made on tiny fragment of “tumour” tissue in needle core biopsies. In an effort to minimise the tissue requirements, we have used multiplex immunofluorescence (MxIF, Cell Dive™) to identify cellular parameters associated with BCE.

The current study is based on analysis of a single section of the tissue microarray (TMA) from patients with DCIS. This is a potential limitation of the study in so far as DCIS is a heterogeneous disease and a TMA core does not adequately represent the extent of disease. This may also explain why data from at least 10 patients could not be used in the final analysis due to absence of DCIS lesions in the section. That being said, this is an exploratory study on cell protein phenotypes and TMAs provide a convenient approach to analyse a larger number of patients. Thirty-two markers were analysed on a single paraffin section using 15 cycling rounds of staining and imaging. This is a major strength of the study. However, the analysis also resulted in data loss due to the requirement that field be composed almost entirely of DCIS cells. This criterion was used to reduce the impact of normal (contaminating) epithelial elements and made it easier to analyse the data as it did not require cell-level classification of the lesions. Further improvement of the analysis algorithms, such as machine learning based approaches for sub-regions of interest, would likely reduce such losses. Further validation studies to confirm the accuracy and reliability of this prognostic signature are necessary and are being planned.

The association of grade with recurrence was non-significant. However, it must be noted that grade was not significant in the analysis of 108,196 patients from the SEER database in multivariate analysis.^36^ In univariate analysis, only ER, PR, and HER2 were associated with likelihood of BCE. This is consistent with prior literature and suggests that the result observed herein can be potentially generalisable. None of the other markers analysed, including grade were associated with BCE. This may be due to small sample size, and insufficient power to detect additional prognostic role of features with a weaker influence on outcome. The expression of both Ki67 and COX2, which in the context of DCIS has been associated with proliferation,^9^ was not associated with BCE. These results are in contrast to prior studies, where Ki67 and COX2 expression were associated with recurrence.^18^ Importantly, mRNAs of the proliferation related genes play an important role in Oncotype Dx DCIS score.^19^ One possible explanation of these differences is that we used an automated quantitation method of signal intensity for all cells included in the analysis and our final unit of measurement for each cell was median nuclear intensity. While all levels of nuclear intensity are considered positive in the assessment of Ki67,^38,39^ we could not apply a pathologist determined cut-off and unsupervised analysis of biomarker intensities versus outcome was conducted. Furthermore, Ki67 levels in DCIS are low and are not ‘thresholded’ in the DCIS score, in contrast to the 21-gene recurrence score.^19^

Although the expression of SLC7A5 and cMET was not significant in univariate analysis, cluster 4 with high expression of cMET, moderate/high ER, low HER2 and SLC7A5 was associated with low risk of BCE. However, cluster 5 and 6, with high HER2 expression resulted in increased risk of BCE. Of note, cMET and SLC7A5 have not been previously implicated in prognostication of DCIS. In invasive cancer, cMET overexpression is seen in metastatic tumours and the amino acid transporter SLC7A5 is a component of the MammaStrat™ signature and more recently shown to be a key therapeutic target in ER+ breast cancer.^40^ These data support the relevance of cMET and SLC7A5 proteins in the biology of breast cancer.

The combination of cluster-based expression scores of ER/HER2/cMET and SLC7A5 markers contributed to development of the Escore algorithm, which was significantly predictive of likelihood of BCE (*P* = 0.00005). In preliminary validation using leave one out cross-validation (LOOCV) method, Escore remained significant (*P* = 0.006). In addition, analysis of duplicate cores from the same patients resulted in similar Escores.

There are a number of limitations to the current study. The analysis was based on retrospective cohort with negative margins (margins status could not be reconfirmed). The analysis was also based on ‘median cell intensity’ and ‘median nuclear intensity’ and the cut-offs were not optimised in the current study. This could have possibly caused lack of significant association of some biomarkers, such as Ki67 and Cox2 with BCE. Lastly, the study is exploratory in nature and the findings need further validation in additional cohorts to understand the value of spatial multiplex immunofluorescence analysis and utility of the Escore. However, we believe that the analysis at single cell level provides considerable strengths and the development of Escore is based on the expression of biomarkers known to play important role in breast cancer. It is important to note that the cohort used herein was matched for treatment. There was no difference in treatment regimens between the BCE and non-BCE patients, who were split approximately 50/50 for treatment and non-treatment regimens (Fig. 2). Further analyses will include replication of the algorithm using Cell Dive™ that necessitates use of multiple markers for cell segmentation as well simpler methods using just the four markers and cell-based analysis. Success in generating the Escore using simple(r) IHC methods could result in rapid dissemination of the results and their implementation in clinical practice.

## Supplementary information

Supplemental materials combined

## Data Availability

Additional information can be found in Supplemental data section. The datasets used and/or analysed during the current study are available from the corresponding author on reasonable request.

## References

[1] Ebctcg, McGale P, Taylor C, Correa C, Cutter D, Duane F (2014). Effect of radiotherapy after mastectomy and axillary surgery on 10-year recurrence and 20-year breast cancer mortality: meta-analysis of individual patient data for 8135 women in 22 randomised trials. Lancet.

[2] Cuzick J, Sestak I, Pinder SE, Ellis IO, Forsyth S, Bundred NJ (2011). Effect of tamoxifen and radiotherapy in women with locally excised ductal carcinoma in situ: long-term results from the UK/ANZ DCIS trial. Lancet Oncol..

[3] Wapnir IL, Dignam JJ, Fisher B, Mamounas EP, Anderson SJ, Julian TB (2011). Long-term outcomes of invasive ipsilateral breast tumor recurrences after lumpectomy in NSABP B-17 and B-24 randomized clinical trials for DCIS. J. Natl. Cancer Inst..

[4] Sanders ME, Schuyler PA, Dupont WD, Page DL (2005). The natural history of low-grade ductal carcinoma in situ of the breast in women treated by biopsy only revealed over 30 years of long-term follow-up. Cancer.

[5] Jones JL (2006). Overdiagnosis and overtreatment of breast cancer: progression of ductal carcinoma in situ: the pathological perspective. Breast Cancer Res..

[6] Collins LC, Tamimi RM, Baer HJ, Connolly JL, Colditz GA, Schnitt SJ (2005). Outcome of patients with ductal carcinoma in situ untreated after diagnostic biopsy: results from the Nurses’ Health Study. Cancer.

[7] Elshof LE, Tryfonidis K, Slaets L, van Leeuwen-Stok AE, Skinner VP, Dif N (2015). Feasibility of a prospective, randomised, open-label, international multicentre, phase III, non-inferiority trial to assess the safety of active surveillance for low risk ductal carcinoma in situ—The LORD study. Eur. J. Cancer.

[8] Fallowfield L, Francis A, Catt S, Mackenzie M, Jenkins V (2012). Time for a low-risk DCIS trial: harnessing public and patient involvement. Lancet Oncol..

[9] Groen EJ, Elshof LE, Visser LL, Rutgers EJT, Winter-Warnars HAO, Lips EH (2017). Finding the balance between over- and under-treatment of ductal carcinoma in situ (DCIS). Breast.

[10] Grimm LJ, Ryser MD, Partridge AH, Thompson AM, Thomas JS, Wesseling J (2017). Surgical Upstaging rates for vacuum assisted biopsy proven DCIS: implications for active surveillance trials. Ann. Surg. Oncol..

[11] Badve S, A’Hern RP, Ward AM, Millis RR, Pinder SE, Ellis IO (1998). Prediction of local recurrence of ductal carcinoma in situ of the breast using five histological classifications: a comparative study with long follow-up. Hum. Pathol..

[12] Badve S, Gokmen-Polar Y (2015). Tumor heterogeneity in breast cancer. Adv. Anat. Pathol..

[13] Gerdes MJ, Gokmen-Polar Y, Sui Y, Pang AS, LaPlante N, Harris AL (2018). Single-cell heterogeneity in ductal carcinoma in situ of breast. Mod. Pathol..

[14] Harrison BT, Hwang S, Partridge A, Thompson A, Schnitt SJ (2018). Variability in diagnostic threshold for comedo necrosis among pathologists: implications for patient eligibility for active surveillance trials of DCIS. Mod. Pathol..

[15] Solin LJ, Gray R, Hughes LL, Wood WC, Lowen MA, Badve SS (2015). Surgical excision without radiation for ductal carcinoma in situ of the breast: 12-year results from the ECOG-ACRIN E5194 study. J. Clin. Oncol..

[16] Sagara Y, Mallory MA, Wong S, Aydogan F, DeSantis S, Barry WT (2015). Survival benefit of breast surgery for low-grade ductal carcinoma in situ: a population-based cohort study. JAMA Surg..

[17] Kerlikowske K, Molinaro AM, Gauthier ML, Berman HK, Waldman F, Bennington J (2010). Biomarker expression and risk of subsequent tumors after initial ductal carcinoma in situ diagnosis. J. Natl. Cancer Inst..

[18] Bremer T, Whitworth PW, Patel R, Savala J, Barry T, Lyle S (2018). A biological signature for breast ductal carcinoma in situ to predict radiotherapy benefit and assess recurrence risk. Clin. Cancer Res..

[19] Solin LJ, Gray R, Baehner FL, Butler SM, Hughes LL, Yoshizawa C (2013). A multigene expression assay to predict local recurrence risk for ductal carcinoma in situ of the breast. J. Natl. Cancer Inst..

[20] Rakovitch, E., Nofech-Mozes, S., Hanna, W., Sutradhar, R., Baehner, F. L., Miller, D. P. et al. Multigene expression assay and benefit of radiotherapy after breast conservation in ductal carcinoma in situ. *J Natl Cancer Inst*. **109**, djw256 (2017). 10.1093/jnci/djw256 PMC6233855 30053207

[21] Gokmen-Polar Y, Nakshatri H, Badve S (2011). Biomarkers for breast cancer stem cells: the challenges ahead. Biomark. Med..

[22] Wright HJ, Hou J, Xu B, Cortez M, Potma EO, Tromberg BJ (2017). CDCP1 drives triple-negative breast cancer metastasis through reduction of lipid-droplet abundance and stimulation of fatty acid oxidation. Proc. Natl. Acad. Sci. USA.

[23] Yang C, He P, Liu Y, He Y, Yang C, Du Y (2015). Down-regulation of CEACAM1 in breast cancer. Acta Biochim. Biophys. Sin. (Shanghai)..

[24] Wakasugi E, Kobayashi T, Tamaki Y, Ito Y, Miyashiro I, Komoike Y (1997). p21(Waf1/Cip1) and p53 protein expression in breast cancer. Am. J. Clin. Pathol..

[25] Nasir A, Holzer TR, Chen M, Man MZ, Schade AE (2017). Differential expression of VEGFR2 protein in HER2 positive primary human breast cancer: potential relevance to anti-angiogenic therapies. Cancer Cell Int..

[26] Hicks DG, Janarthanan BR, Vardarajan R, Kulkarni SA, Khoury T, Dim D (2010). The expression of TRMT2A, a novel cell cycle regulated protein, identifies a subset of breast cancer patients with HER2 over-expression that are at an increased risk of recurrence. BMC Cancer.

[27] Bartlett JM, Thomas J, Ross DT, Seitz RS, Ring BZ, Beck RA (2010). Mammostrat as a tool to stratify breast cancer patients at risk of recurrence during endocrine therapy. Breast Cancer Res..

[28] Kochel TJ, Reader JC, Ma X, Kundu N, Fulton AM (2017). Multiple drug resistance-associated protein (MRP4) exports prostaglandin E2 (PGE2) and contributes to metastasis in basal/triple negative breast cancer. Oncotarget.

[29] Mao Q, Unadkat JD (2015). Role of the breast cancer resistance protein (BCRP/ABCG2) in drug transport–an update. AAPS J..

[30] Delou JMA, Vignal GM, Indio-do-Brasil V, Accioly MTS, da Silva TSL, Piranda DN (2017). Loss of constitutive ABCB1 expression in breast cancer associated with worse prognosis. Breast Cancer (Dove Med Press)..

[31] Gerdes MJ, Sevinsky CJ, Sood A, Adak S, Bello MO, Bordwell A (2013). Highly multiplexed single-cell analysis of formalin-fixed, paraffin-embedded cancer tissue. Proc. Natl Acad. Sci. USA..

[32] Wilkerson MD, Hayes DN (2010). ConsensusClusterPlus: a class discovery tool with confidence assessments and item tracking. Bioinformatics.

[33] Șenbabaoğlu Y, Michailidis G, Li JZ (2014). Critical limitations of consensus clustering in class discovery. Sci. Rep..

[34] Cardoso F, van’t Veer LJ, Bogaerts J, Slaets L, Viale G, Delaloge S (2016). 70-Gene signature as an aid to treatment decisions in early-stage breast cancer. N. Engl. J. Med..

[35] Sparano JA, Gray RJ, Makower DF, Pritchard KI, Albain KS, Hayes DF (2018). Adjuvant chemotherapy guided by a 21-gene expression assay in breast cancer. N. Engl. J. Med..

[36] Narod SA, Iqbal J, Giannakeas V, Sopik V, Sun P (2015). Breast cancer mortality after a diagnosis of ductal carcinoma in situ. JAMA Oncol..

[37] Esserman LJ, Thompson IM, Reid B, Nelson P, Ransohoff DF, Welch HG (2014). Addressing overdiagnosis and overtreatment in cancer: a prescription for change. Lancet Oncol..

[38] Leung SCY, Nielsen TO, Zabaglo L, Arun I, Badve SS, Bane AL (2016). Analytical validation of a standardized scoring protocol for Ki67: phase 3 of an international multicenter collaboration. NPJ Breast Cancer.

[39] Leung SCY, Nielsen TO, Zabaglo LA, Arun I, Badve SS, Bane AL (2019). Analytical validation of a standardised scoring protocol for Ki67 immunohistochemistry on breast cancer excision whole sections: an international multicentre collaboration. Histopathology.

[40] El Ansari R, Craze ML, Miligy I, Diez-Rodriguez M, Nolan CC, Ellis IO (2018). The amino acid transporter SLC7A5 confers a poor prognosis in the highly proliferative breast cancer subtypes and is a key therapeutic target in luminal B tumours. Breast Cancer Res..

