# Supplementary material for: Multi-protein spatial signatures in ductal carcinoma in situ (DCIS) of breast
Source: Br J Cancer. 2021 Jan 7;124(6):1150–9. doi: 10.1038/s41416-020-01216-6 (PMC7961015; doi:10.1038/s41416-020-01216-6)
Supplement: Supplementary file 1 — Supplemental materials combined [file 41416_2020_1216_MOESM1_ESM.pdf]

SUPPLEMENTAL INFORMATION

Supplemental Information includes four figures and tables.

Supplementary Data

Supplementary Figure 1: Representative figures showing the expression of individual markers in example DCIS cases.

Supplementary Figure 2: Kaplan Meier plots of entire patient group (n=135) and study cohort included in the MxIF and outcome analysis (n=51).

Supplementary Figure 3: Correlation plots for all cell biomarker data in tumor and stroma.

Supplementary Figure 4: Violin plots showing the association between marker expression and BCE.

Supplementary Table 1. Clinical Demographic Summary

Supplementary Table 2: Antibodies, Clone, and Conjugate Information

Supplementary Table 3: Details of the antibodies and the round of staining used for each marker during the MxIF staining.

Supplementary Table 4: Detailed stepwise flow chart of the analysis and the filters used in the study.

Supplementary Figure 1

Staining Round 1: CK5/6; case 115; FOV 363<br>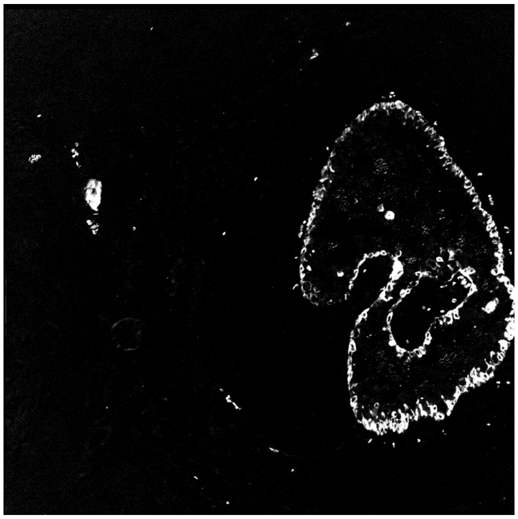	Staining Round 1: Her4; case 115; FOV 363<br>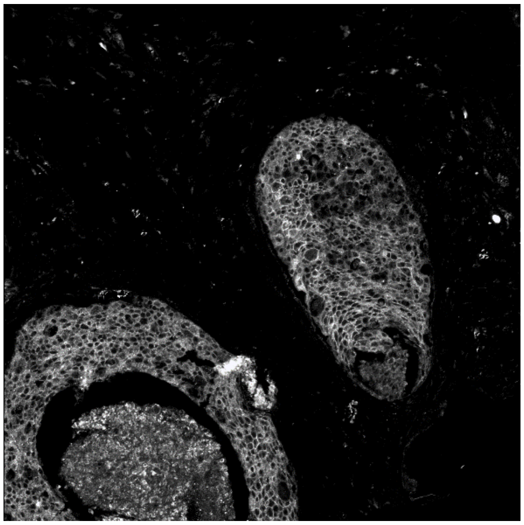
Round 2: ABCG2; case 8; case 115; FOV 363<br>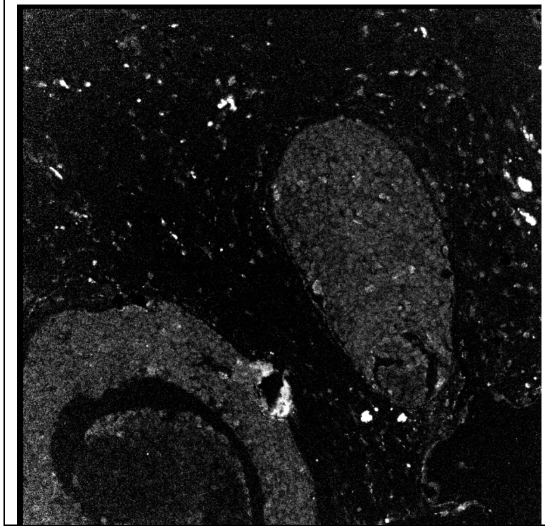	Round 2: PTEN; case 8; case 115; FOV 363<br>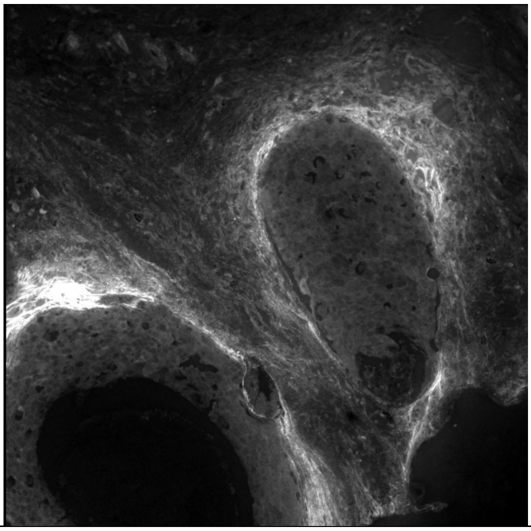

Supplementary Figure 1

Round 3: CD20; Case 363; FOV 115

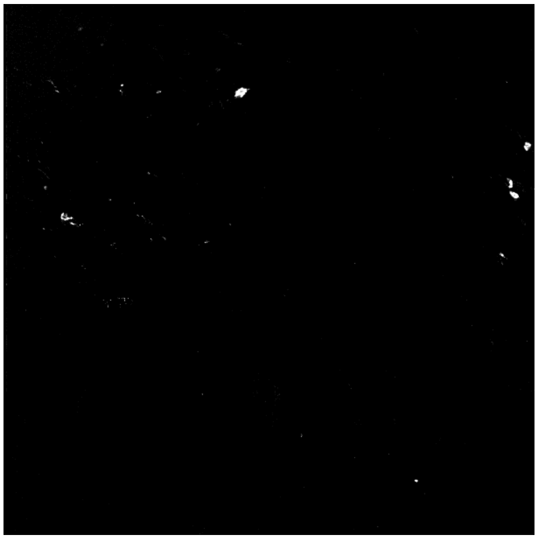

Round 3: S6; Case 363; FOV 115

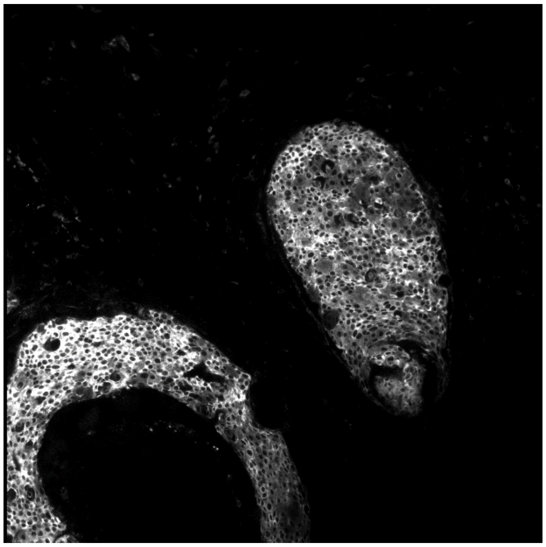

Round 3: CKAE1 Case 363; FOV 115

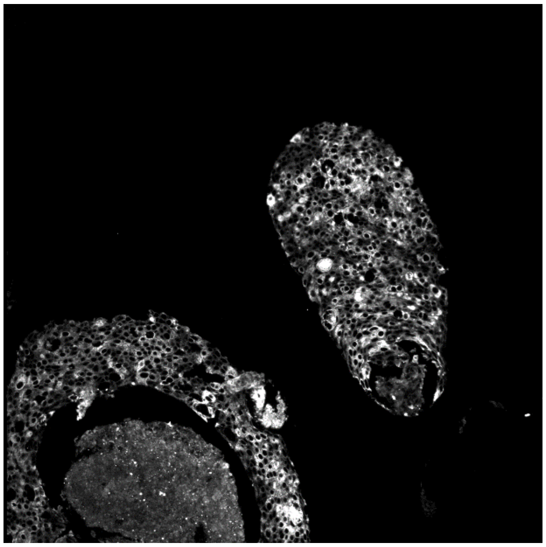

Round 4: PR; Case 363; FOV 11

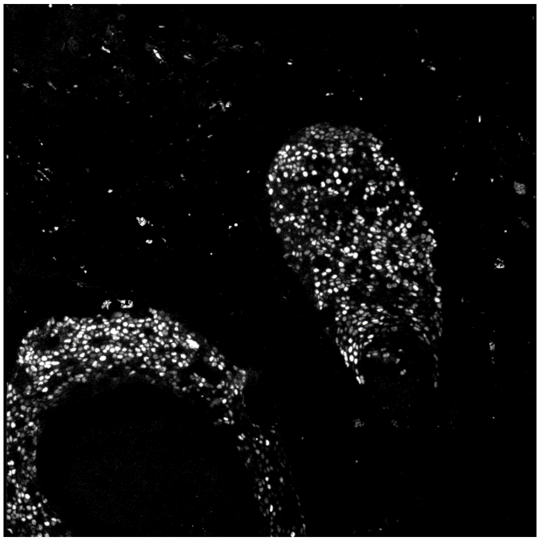

Supplementary Figure 1

Round 4: ER Case 363; FOV 115

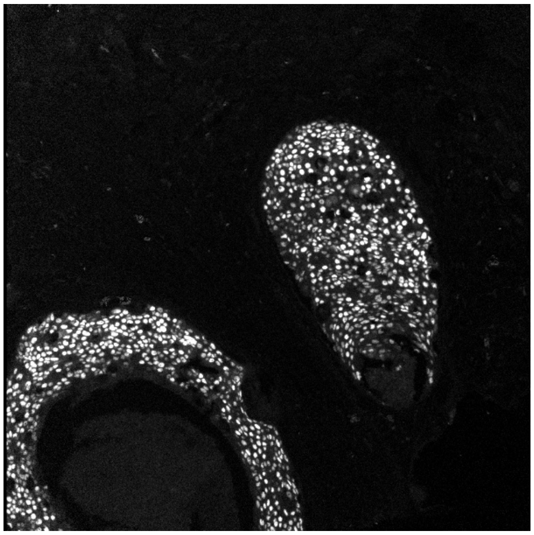

Round 4: NaKATPase Case 363; FOV 115

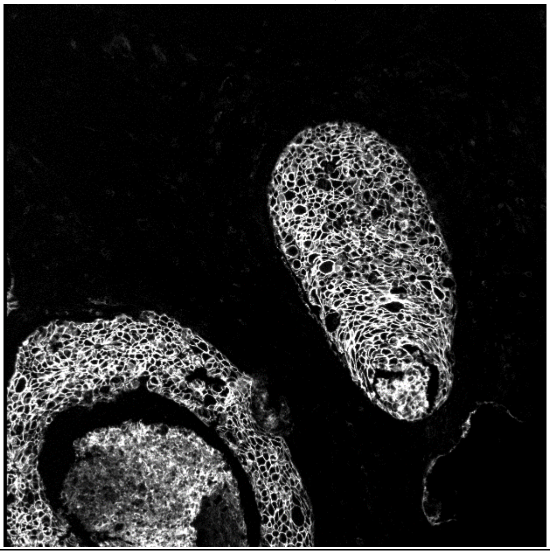

Round 5: CK19 Case 363; FOV 115

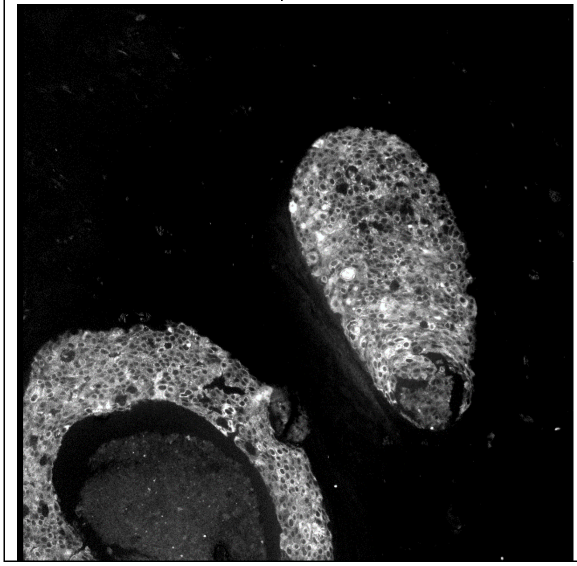

Round 5: ALDH1 Case 363; FOV 115

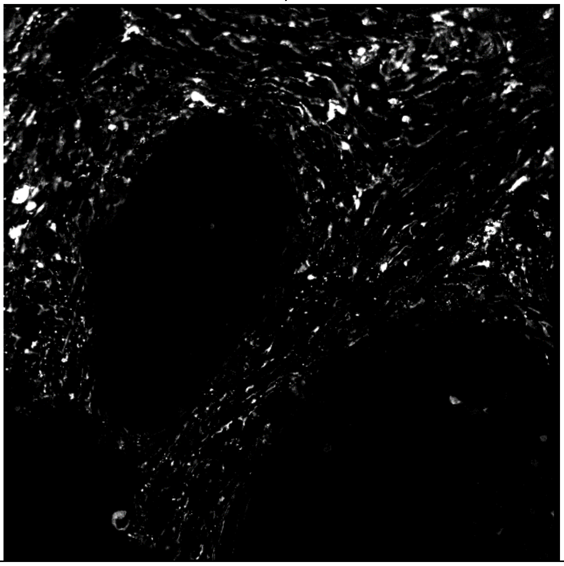

Supplementary Figure 1

Round 5 PCK26 Case 363; FOV 115

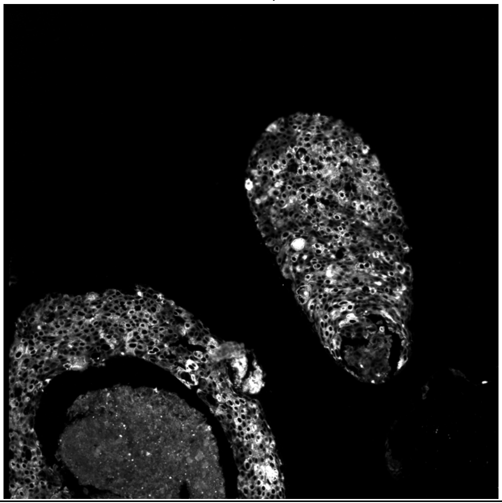

Round 6 CD4 Case 363; FOV 112

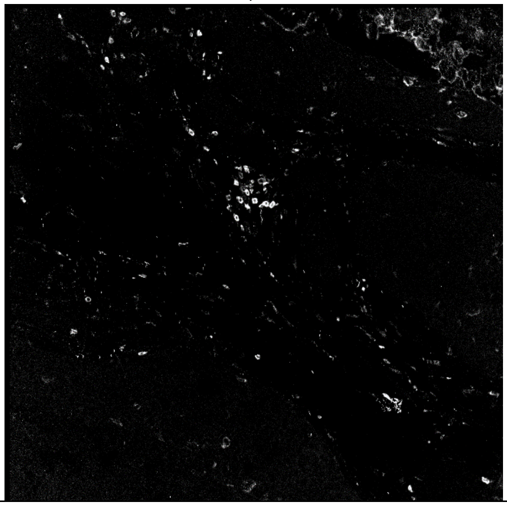

Round 6 cMET Case 363; FOV 115

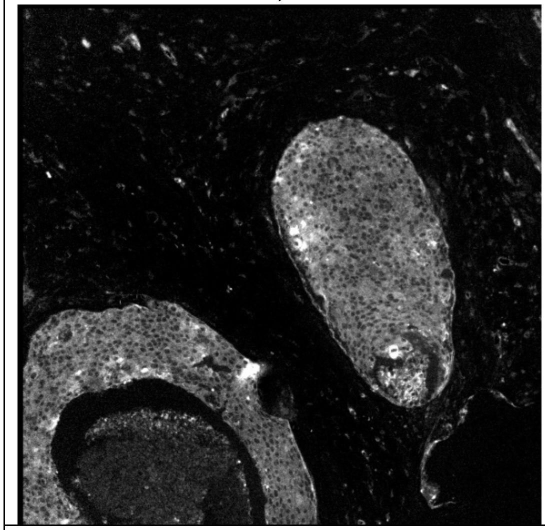

Round 7 CD44v6 Case 363; FOV 95

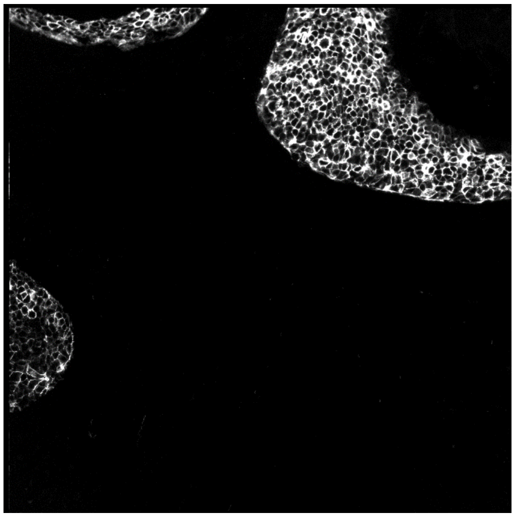

Round 7 Her2 Case 363; FOV 115

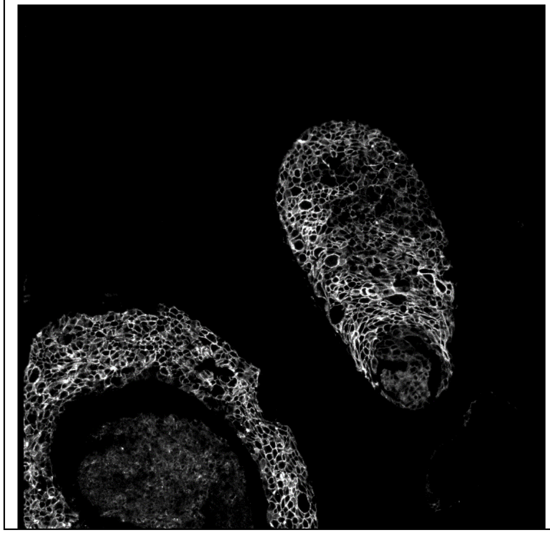

Round 8 CDCP1 Case 363; FOV 112

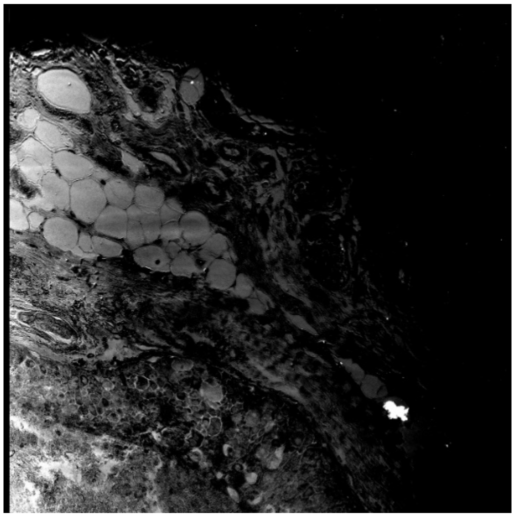

Supplementary Figure 1

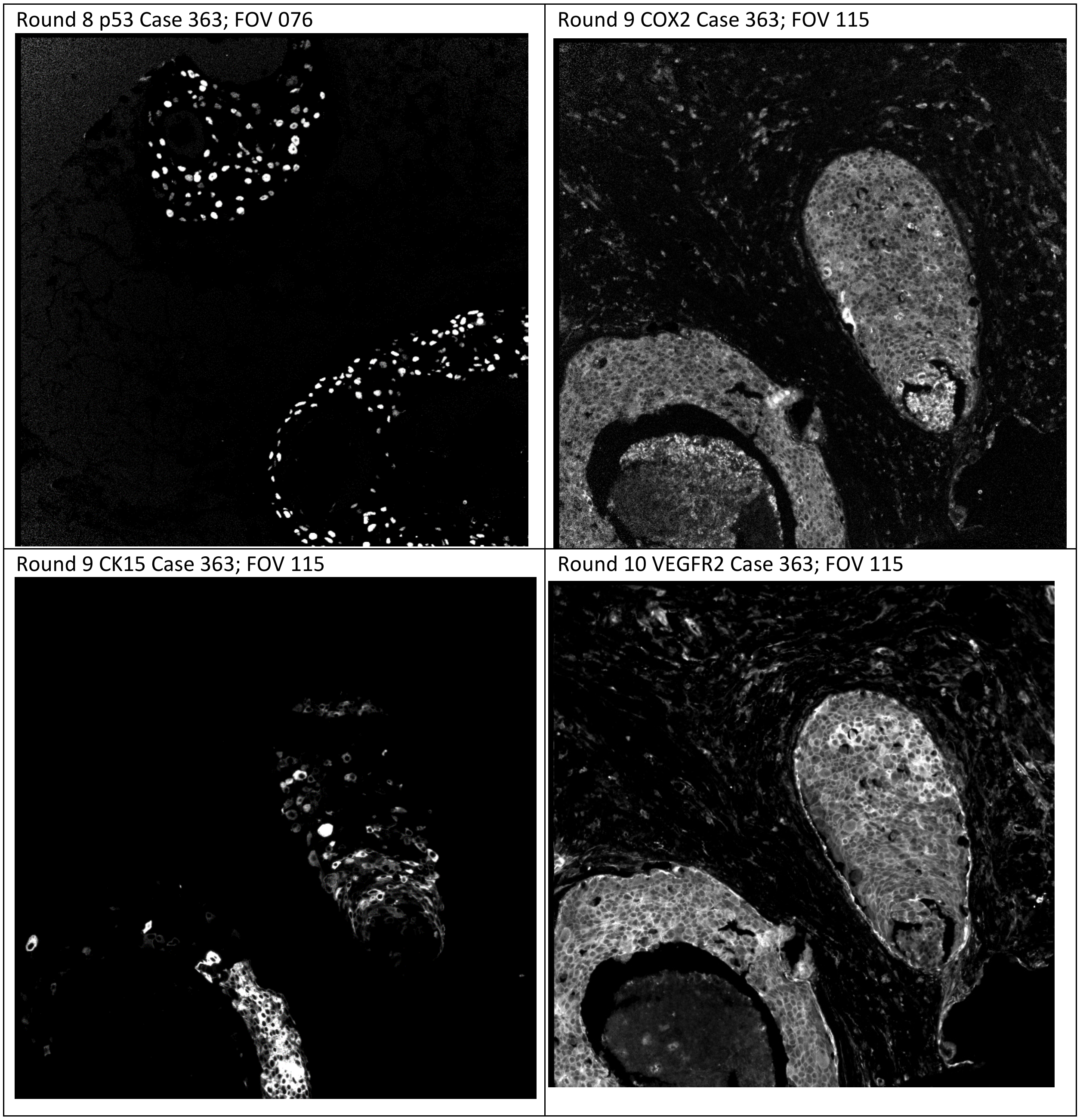

Supplementary Figure 1

Round 10 ABCB1 Case 363; FOV 66

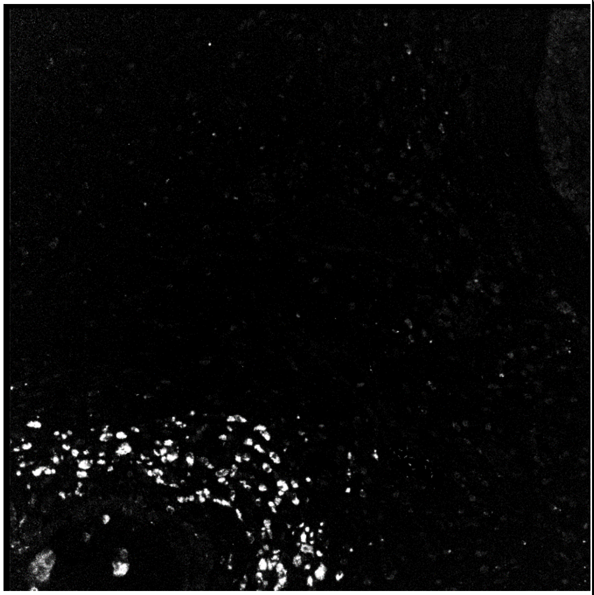

Round 11 CD10 Case 363; FOV 115

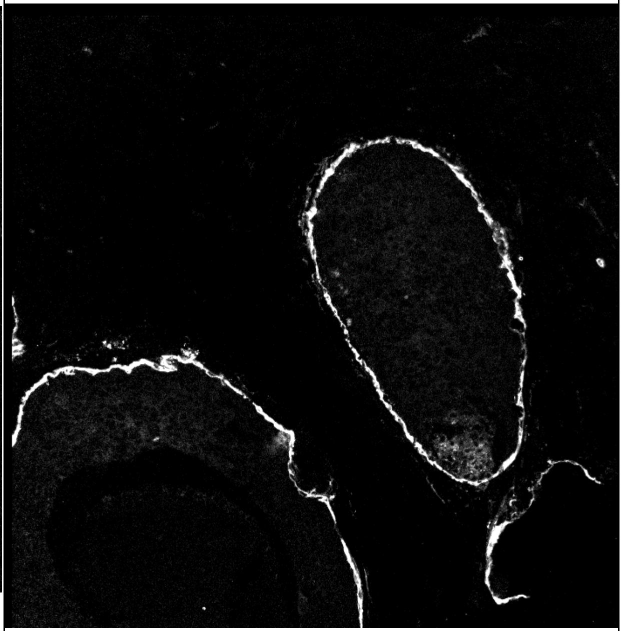

Round 11 HTF9c Case 363; FOV 76

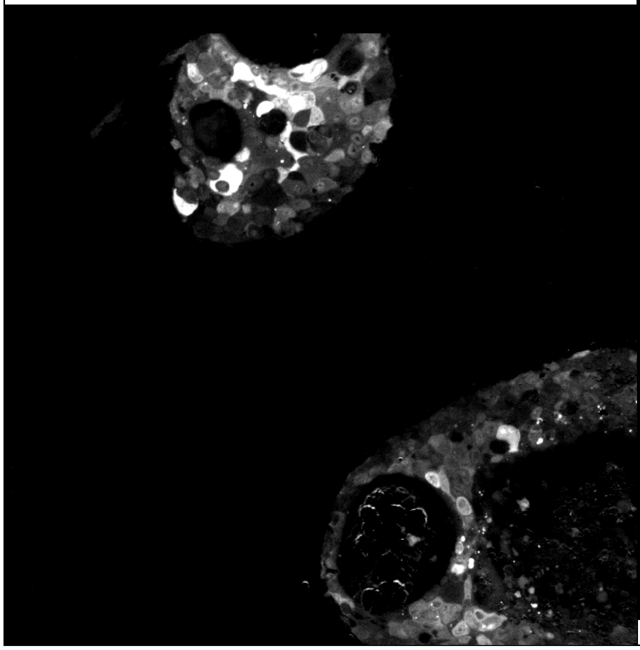

Round 12 SLC7A5 Case 363; FOV 115

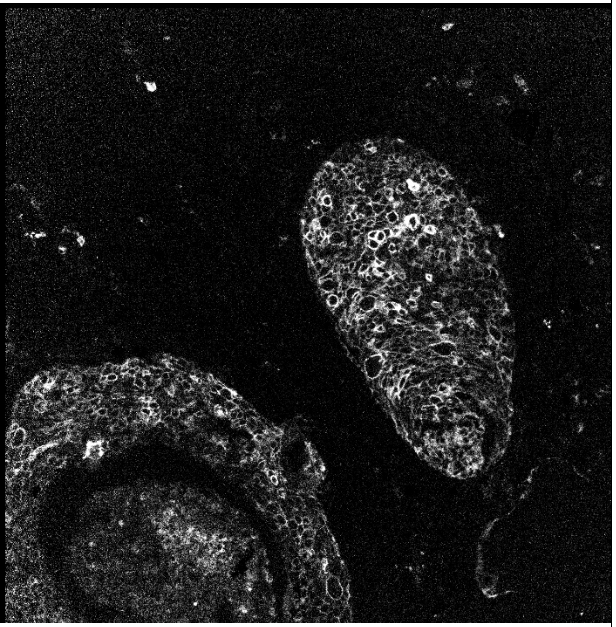

Supplementary Figure 1

Round 12 MRP4 Case 310; FOV 27 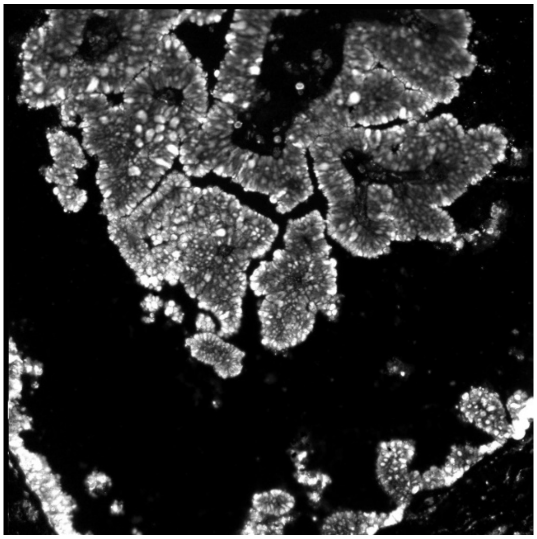	Round 13 p21 Case 363; FOV 115 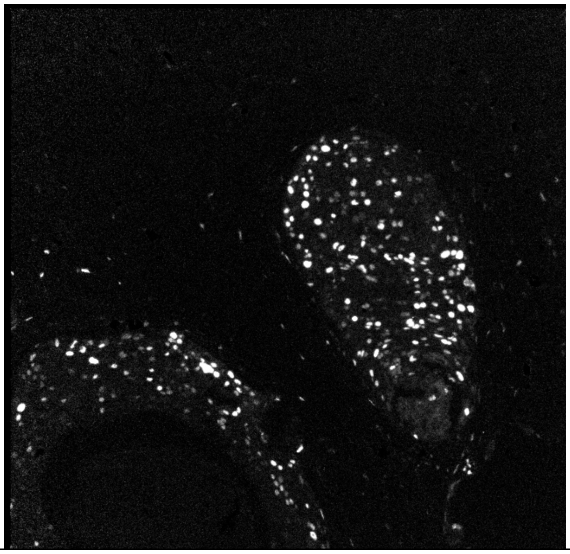
Round 13 EGFR Case 324; FOV 5 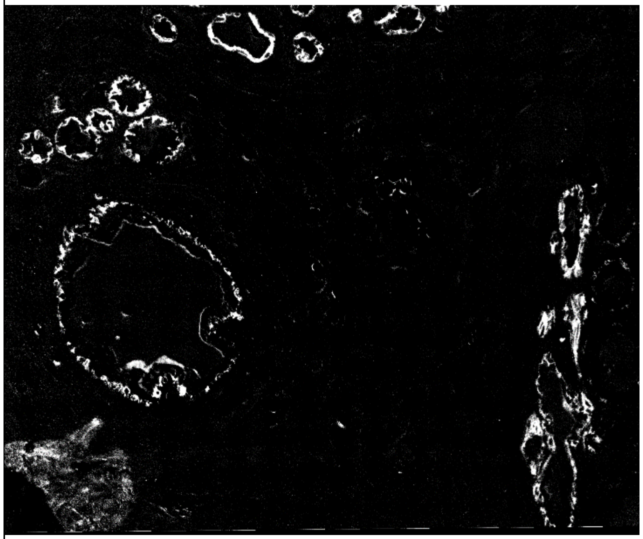	Round 14 CEACAM5 Case 363; FOV 92 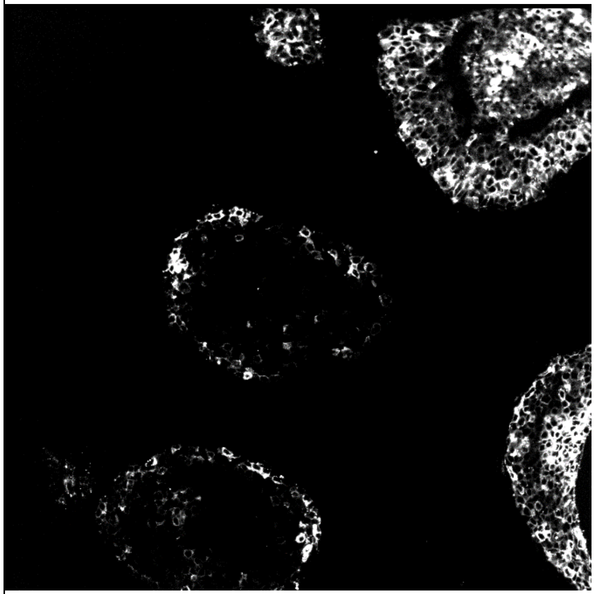

Supplementary Figure 1

Round 14 MRP5 Case 363; FOV 115<br>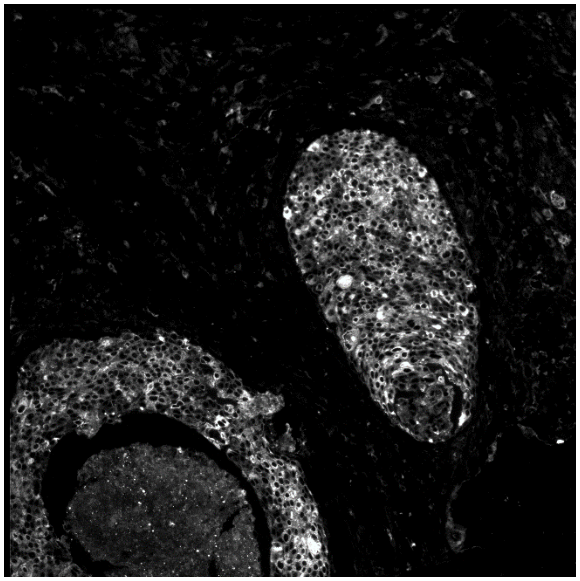	Round 15 Ki67 Case 363; FOV 115<br>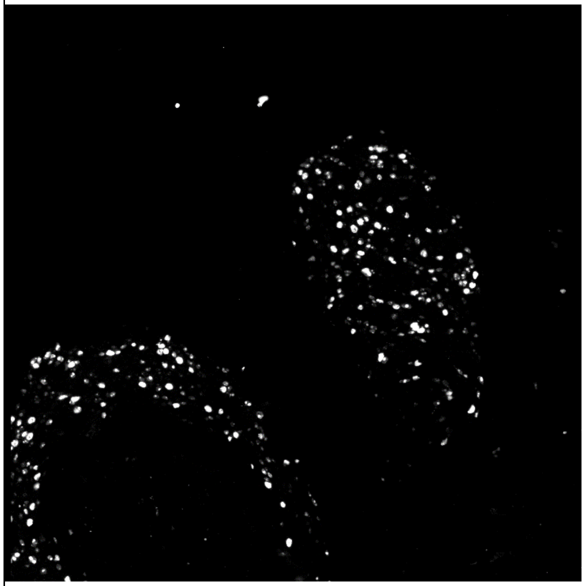

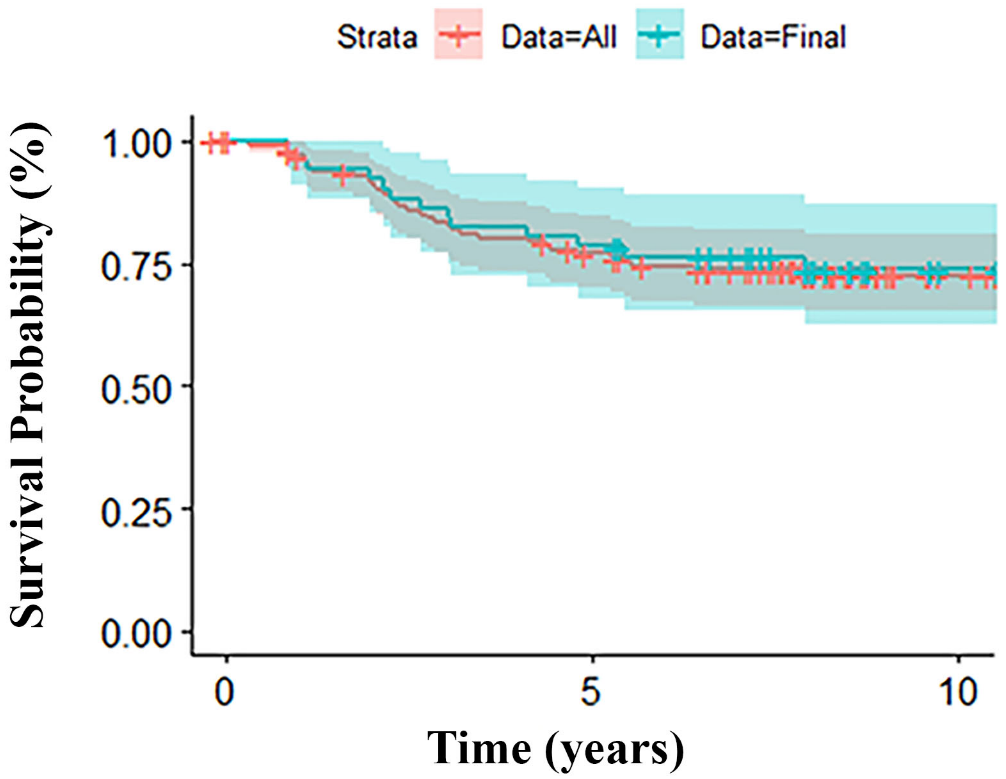

Supplementary Figure 2

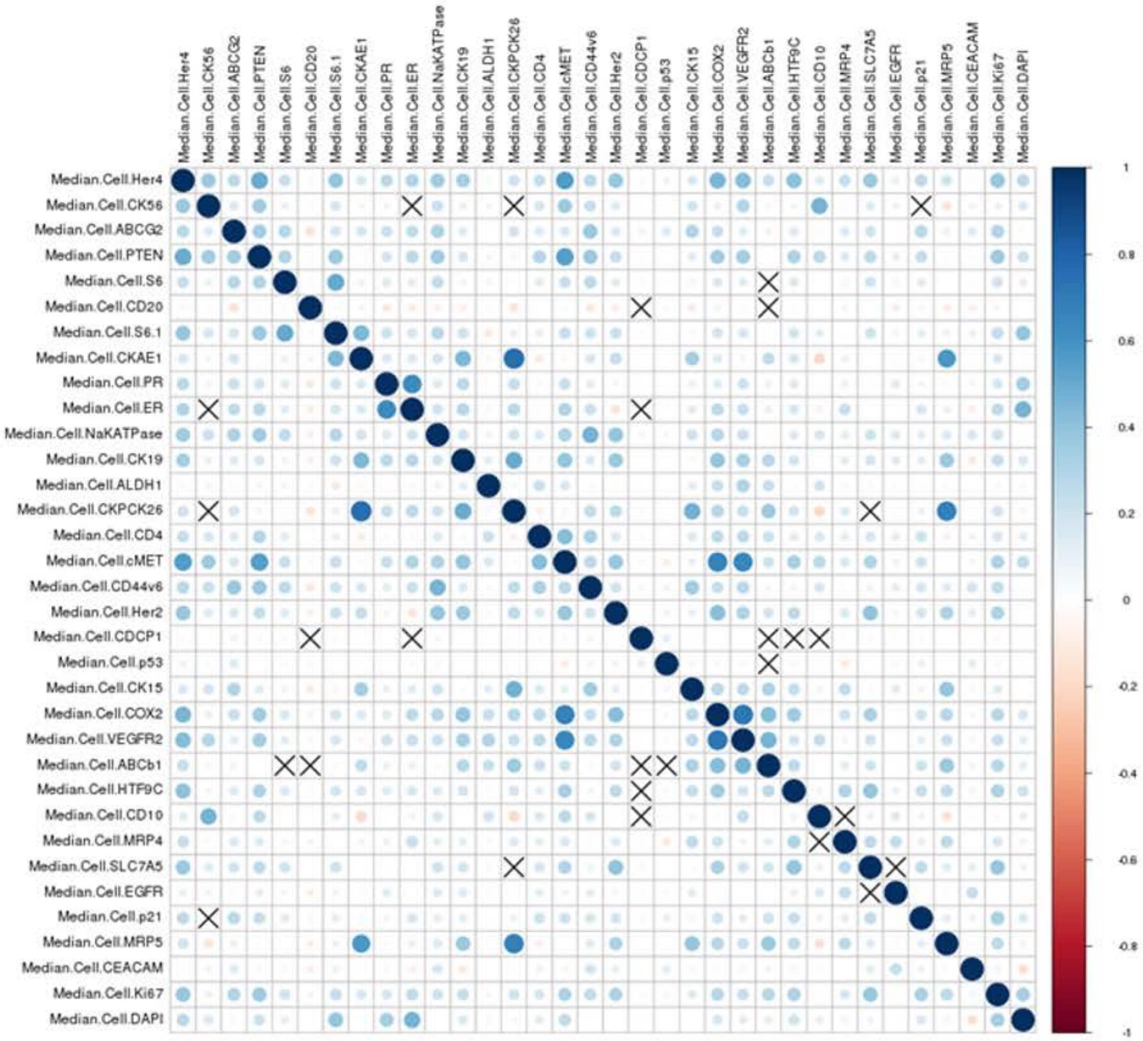

Supplementary Figure 3

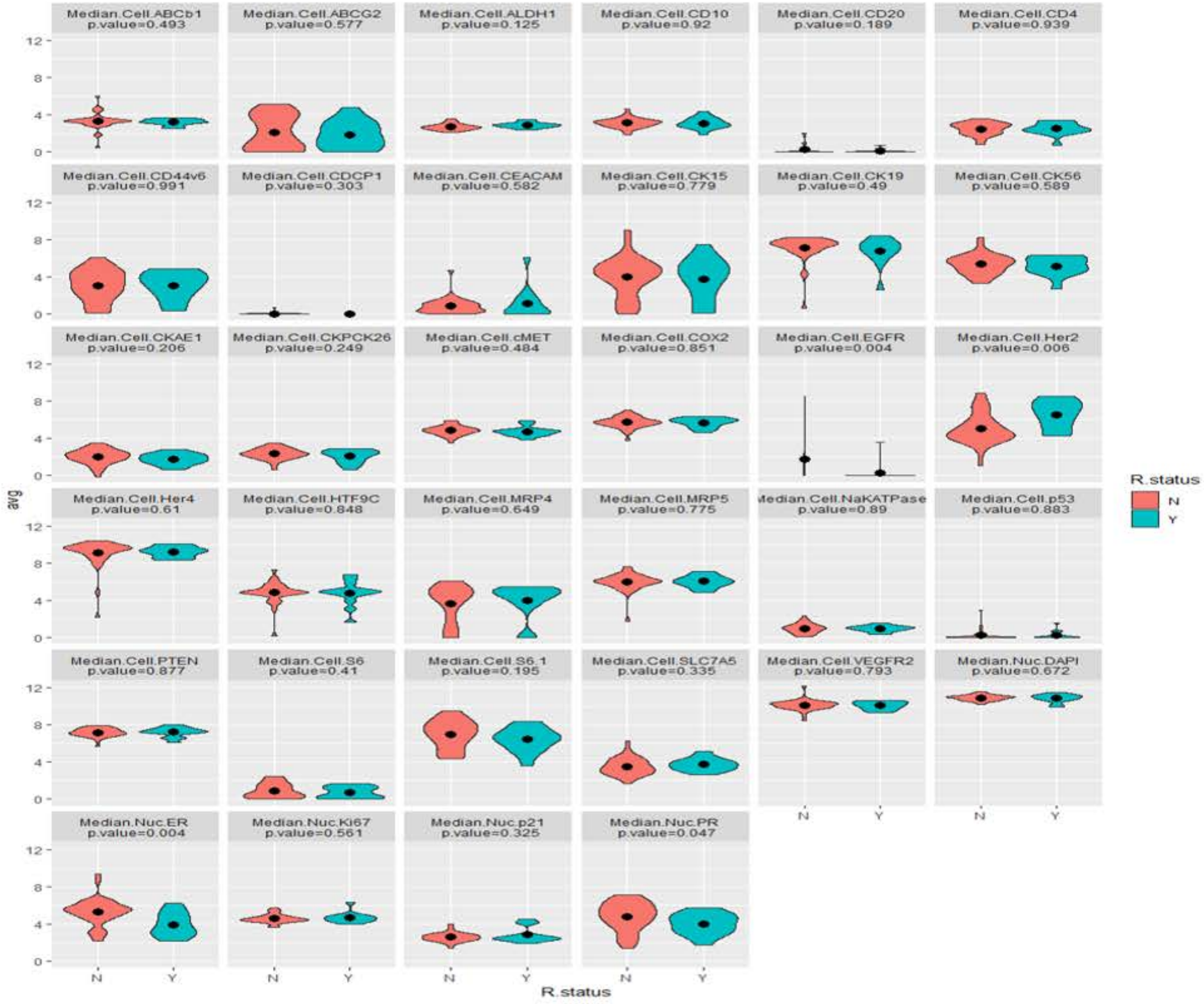

Supplementary Figure 4

Supplementary Table 1. Demographic and Treatment Summary of DCIS patients

	Entire Cohort (n=135)	Study Cohort (n=51)
Age (years)	32 – 75 (median = 55)	34 – 75 (median = 55)
Year of diagnosis	1986 – 2004	1986-1999
DCIS grade (High/Moderate/Low/unknown)	65/44/19/7	26/18/7
Size of DCIS (cm)	0.4 – 9.5 (median = 1.6)	0.6 – 9.5 (median = 2)
Estrogen receptor status (pos/neg/unknown)	20/13/102	13/8/30
Follow up time (years)	0.3– 17 (median = 8)	0.8 – 17 (median = 8)
Recurrent Patients (years)	0.8 – 15 (median = 3)	0.8 – 8 (median = 2.5)*
Non-recurrent Patients (years)	0.3 – 17 (median = 9)	5 – 17 (median = 9)**
Hormone Therapy	44	22
Radiation Therapy	22	13
Hormone and Radiation Therapy	14	8
Breast Cancer event (BCE)	39 (29%)	13 (25%)
In situ	15 (11%)	5 (10%)
Ipsilateral invasive	15 (11%)	6 (12%)
Contralateral invasive	5 (4%)	1 (2%)
Metastatic	4 (3%)	1 (2%)

*Excludes patients with BCE > 10 yrs; **Excludes patients with no BCE < 3 yrs

Supplementary Table 2. Antibodies, Clone, and Conjugate Information

Antibody-Cy3	Supplier/Clone	Antibody-Cy5	Supplier/Clone	Antibody-Cy7	Supplier/Clone
Her4	LS Bio B5500	CK 5/6	Dako M7237		
ABCG2	Millipore MAB4146	PTEN	Cell Signaling 9188	S6	Cell Signaling 2217
CD20	Epitomics 1632	S6	Cell Signaling 2217	CK AE1	eBioscience 14-9001
PR	Dako - M3568	ER	Leica PA0151	NaKATPase	Epitomics 2047
CK19	eBioscience 14-9898	ALDH1	BD-Bio 611195	CK PCK26	Sigma C1801
CD4	Epitomics 5636	C-Met	Cell Signaling 8198P	-	-
CD44v6	eBioscience BMS116	Her2	Cell Signaling 4290	-	-
CDCP1	Cell Signaling 4115	p53	Dako M7001	-	-
CK15	Sigma HPA023910	Cox-2	Invitrogen 35-8200	-	-
VEGFR2	Cell Signaling 2479	MDR1/ABCB1	Biorbyt Orb122787	-	-
HTF9C	Clarient S0729	CD10	Leica NCL-L-CD10-270	-	-
MRP4	Sigma SAB2500011	SLC7A5	AGI S0730	-	-
EGFR	Cell Signaling 5108	p21	Cell Signaling 2947	-	-
MRP5	Abcam Ab24107	CEACAM5	AGI S5092M	-	-
Ki67	Thermo Fisher RB-1510-PABX	-	-	-	-

Supplementary Table 3. Multiplexed Immunofluorescence Staining Sequence for all antibodies.

step	Antibody-Cy3	Staining Concentration	Antibody-Cy5	Staining Concentration	Antibody-Cy7	Staining Concentration
0	Background					
1	Her4	4 g/ml	CK 5/6	10ug/ml		
2	Background					
3	ABCG2	10ug/ml	PTEN	10ug/ml	S6	10ug/ml
4	Background					
5	CD20	2.5ug/ml	S6	5ug/ml	CK AE1	2.5ug/ml
6	Background					
7	PR	10ug/ml	ER	10ug/ml	NaKATPase	5ug/ml
8	Background					
9	CK19	5ug/ml	ALDH1	10ug/ml	CK PCK26	2.5ug/ml
10	Background					
11	CD4	10 µg/mL	C-Met	15ug/ml		
12	CD44v6	5ug/ml	Her2	5ug/ml		
13	CDCP1	20ug/ml	p53	1ug/ml		
14	CK15	5ug/ml	Cox-2	20 µg/mL		
15	VEGFR2	10ug/ml	MDR1/ABCB1	5ug/ml		
16	Background					
17	HTF9C	5ug/ml	CD10	10ug/ml		
18	MRP4	2.5ug/ml	SLC7A5	5ug/ml		
19	EGFR	1ug/ml	p21	5ug/ml		
20	MRP5	5ug/ml	CEACAM5	2.5ug/ml		
21	Ki67	5ug/ml	-			

Supplementary Table 4: Analysis procedure flow down patients to cells.

	N slide	N FOV	N cell	N cores	Details
Initial single cell data	8	748	1,972,390	-	
Epithelial cells	8	748	808,200	-	Only Epithelial cells were used for this study
Staining QC	8	346	479,358	-	• Segmentation quality<br>• Staining quality
Cell filter	8	343	155,179	-	• Number of nuclei: 1 or 2<br>• Areas of cell components (nuc, cyt, memb): 10-1500 pixels
Cells for clustering	8	338	131,568	131	QC score filter: Only included cells with excellent correlation score (=1)
	(k-means clustering performed)				
Outcome Analysis	7	168	74,913	66	Figure 1 describes filtering process. In total, 65 cores were filtered out for outcome analysis.<br>• 37 cores were excluded due to insufficient clinical information.<br>• 21 cores were excluded due to insufficient representation of DCIS.<br>▪ Non-DCIS FOV: (%DCIS/(%DCIS+%normal) <0.5<br>▪ Less than 100 cells/patient<br>• 7 cores were excluded due to duration of follow up time
